# Treatment with intravenous pamidronate is a good alternative in case of gastrointestinal side effects or contraindications for oral bisphosphonates

**DOI:** 10.1186/1471-2474-10-86

**Published:** 2009-07-15

**Authors:** Danielle A Eekman, Marijn Vis, Irene EM Bultink, Harm JGM Derikx, Ben AC Dijkmans, Willem F Lems

**Affiliations:** 1 Department of Rheumatology, VU University Medical Center, Amsterdam, the Netherlands; 2 Department of Rheumatology, Jan van Breemen Institute, Amsterdam, the Netherlands

## Abstract

**Background:**

In case of contraindications or intolerance during treatment with oral bisphosphonates (OB), administration of pamidronate intravenously is a widely used alternative.

In this study we compared the effect on change in bone mineral density (BMD) of the spine and hip during long term treatment with pamidronate iv in comparison to OB.

**Methods:**

We studied 61 patients receiving treatment for at least two years. In case of contraindications or intolerance (within 3 months) of an OB, pamidronate iv was started. BMD was measured on a Hologic 4500 and a Lunar DPX-IQ at the spine (L1-L4) and total hip.

**Results:**

Thirty-one patients were enrolled in the OB group and 30 in the intravenous pamidronate group. Mean follow-up duration (SD) was 4.3 (1.3) years. We observed a significant increase (p < 0.001) in spinal BMD, both in the OB group (8.3%) as well as in the pamidronate iv group (6.1%), but no significant difference in BMD change between the OB and pamidronate iv groups. At the hips, we observed a tendency to increased BMD in both groups, 1.1% in the OB and 1.4% in the pamidronate iv group.

**Conclusion:**

We conclude that intravenous pamidronate is a good alternative for oral bisphosphonates in the treatment of osteoporosis in patients with contraindications or intolerance during treatment with oral bisphosphonates.

## Background

In a previous one year prospective study we showed evidence of a similar effectiveness of oral bisphosphonates compared to pamidronate iv. [1] Patients were treated with alendronate 10 mg orally once daily or pamidronate 60 mg intravenously every three months. In both groups the BMD of the lumbar spine as well as the total hip increased equally after one year of treatment. It is uncertain however, whether the effect remains comparable after a longer follow up period.

Treatment with anti-osteoporotic drugs, such as bisphosphonates, has shown to effectively increase bone mineral density (BMD) and reduce vertebral fracture risk by as much as 40–50% [2,3] Fractures are the most important clinical manifestation of osteoporosis and can cause substantial morbidity [4] and mortality [5]. Oral bisphosphonates however, may cause upper gastro intestinal side effects such as dyspepsia and abdominal pain and are contraindicated in case of comorbidity of the upper gastrointestinal tract. [6,7]

In the current study, we observed the change in BMD of lumbar spine and total hip after 2–5 years of treatment with either pamidronate intravenously or oral bisphosphonates.

## Methods

We performed a retrospective study at the osteoporosis outpatient clinic of the Slotervaart Hospital (Amsterdam, the Netherlands). After screening for osteoporosis, treatment with anti-osteoporotic drugs was started, based on: 1) a T score of ≤ -2.5 at the spine and/or hip, 2) one or more vertebral fractures (height loss more than 20%) or 3) a daily dose of prednisone of 7.5 mg or more during at least three months, and a T-score < -1 at the spine or hips, after which every one to two years patients returned to the clinic for a follow up visit.

During the follow up visits, 61 consecutive patients treated for at least two years with either an oral bisphosphonate (alendronate 70 mg weekly or risedronate 35 mg weekly) or intravenous pamidronate (60 mg 3 monthly, dissolved in 250 ml 0.9% saline) were enrolled. At that time it was not unusual to retrospectively and anonymously collect data based on treatment effects of anti-osteoporotic drugs in daily practice, without approval from the ethics board. (all data were collected before approval became mandatory).

The indications for intravenous pamidronate were contraindications for oral bisphosphonates or intolerance for oral bisphosphonate therapy within three months from the start of treatment. Patients with previous treatment for longer than 3 months with anti-osteoporotic drugs were not included.

Demographic data and data on risk factors for osteoporosis at baseline and after a period of 2–5 years were collected. The data collected at baseline included: type of bisphosphonate (oral or pamidronate) and reason for intravenous administration, age, body mass index (BMI), menopausal status, history of clinical fractures (wrist, ankle or hip), family (first degree) history of fractures, dietary intake of calcium, supplementation of calcium and/or vitamin D, history of prednisone use and laboratory testing including erythrocyte sedimentation rate, thyroid stimulating hormone, free T4, creatinine, urea, alkaline phosphatase, calcium and 25(OH) vitamin D.

BMD was measured with dual X-ray absorptiometry (DXA) at the start of treatment and after 2–5 years in the lumbar spine (L1-L4) and the total hip. The majority of the measurements were performed on a Hologic 4500 (Waltham, Mass., USA) and some on a Lunar DPX-IQ (Lunar). Repeated measurements in all patients were done on the same machine.

### Statistical analyses

To compare mean change in BMD between the groups and within the groups we used the independent t-test and paired student's t-test, respectively. The distribution of risk factors and demographic data were compared between groups using student's t-test for continuous variables and using Pearson's chi-square test for dichotomous variables.

## Results

The baseline characteristics of patients treated with pamidronate iv or oral bisphosphonates are shown in table 1. The mean duration of treatment was more than 4 years and comparable in both groups. There were no significant differences between the groups in age, gender, age at menopause, BMI, history of clinical vertebral or nonvertebral fractures, family history of fractures, calcium intake, use of prednisone or serum calcium, albumin or 25(OH) vitamin D level. Laboratory testing showed no abnormalities in thyroid or kidney function. The BMD and T scores of spine and hip at baseline were similar as well.

**Table 1:** Baseline characteristics of patients treated with pamidronate iv or oral bisphosphonates

**Variables**		**Oral Bisphosphonates n = 31**	**Pamidronate n = 30**	p value
Duration of treatment (years)	mean (range)	4.2 (4.9)	4.3 (4.8)	0.914
Age (years)	mean (SD)	62 (13.9)	67 (9.6)	0.132
Gender (female)	n (%)	24 (77)	24 (80)	0.527
Age at menopause (years)	mean (SD)	48 (4.6)	46 (5.3)	0.178
BMI (kg/m2)	mean (SD)	24 (4.7)	25 (4.5)	0.636
				
Number of patients with at least 1 clinical fracture	n (%)	9 (29)	5 (17)	0.251
Familial fractures	n (%)	12 (36)	11 (37)	0.516
				
Dietary calcium intake (mg/day)	mean (SD)	925 (366)	838 (241)	0.293
Calcium supplementation daily dose (mg)	n (%)	12	9	0.678
	mean (SD)	541 (144)	610 (334)	0.531
Treatment with prednisone daily dose (mg)	n (%)	6 (18)	5 (17)	0.835
	mean (SD)	10 (4.2)	11.5 (6)	0.638
				
T score spine	mean (SD)	-2.39 (1.55)	-2.58 (0.89)	0.562
T score hip	mean (SD)	-1.85 (1.13)	-1.79 (1.03)	0.816
				
Serum 25(OH) D levels (nmol/l)	mean (SD)	70 (31)	62 (29)	0.313
Serum calcium levels (mmol/l)	mean (SD)	2.38 (0.1)	2.39 (0.1)	0.678
Serum albumin levels (g/l)	mean (SD)	41 (9)	39 (4)	0.356

At baseline there are no significant differences between the oral bisphosphonates group and the pamidronate iv group

Twenty-one patients (34%) used calcium supplementation because of an insufficient dietary calcium intake (<1000 mg daily), twelve in the oral bisphosphonates group and nine in the pamidronate iv group. A serum 25 (OH) vitamin D level below 30 nmol/l at baseline was an indication to start supplementation with cholecalciferol. Five patients treated with oral bisphosphonates were given supplementation with vitamin D, as well as six in the pamidronate iv group. In the oral bisphosphonates group 17 patients were treated with alendronate 70 mg weekly and 14 with risedronate 35 mg weekly.

During the outpatient clinic visits patients were asked about their adherence to therapy. All patients confirmed that they had taken their pills regularly, one patient missed one infusion pamidronate due to a self-limiting respiratory tract infection. Since it was a retrospective study, and only patients who had tolerated their treatment during at least two years were included, there are hardly any data on side effects available.

In the oral bisphosphonates group 21 out of 31 had a T score of ≤ -2.5 at either spine or hip, 6 were using a high dose of prednisone and 7 had a vertebral fracture. (three patients had both a low T score and were using a high dose of prednisone) In the pamidronte iv group 16 of the 30 had a T score of ≤ -2.5 at either spine or hip, 5 were using a high dose of prednisone and 9 had a vertebral fracture. The T scores by site or fracture history parameters that resulted in study inclusion were the same in both groups.

The indication for treatment with pamidronate iv was intolerance of oral bisphosphonates (22), gastrointestinal complaints prior to treatment with oral bisphosphonates (4) and a contraindication for oral treatment (4).

During the follow up period, three patients in the oral bisphosphonates group and two in the pamidronate group had a new non-vertebral osteoporotic fracture (two ankle fractures and a hip fracture, and two hip fractures, respectively). There were no new clinical vertebral fractures.

### Change in BMD

There was a small tendency to a larger increase of BMD in patients using alendronate, eg in the spine the BMD increased (SD) with 9 (8.7)% versus 7.5 (12)% in patients using risedronate. However, these differences were not significant, therefore further analyses has been performed on all patients using oral bisphosphonates.

The BMD (SD) of the lumbar vertebral spine increased with 0.058 (0.09) g/cm2 (+8.3% p < 0.001 vs baseline) in the oral and 0.048 (0.05) g/cm2 (+6.1% p < 0.001 vs baseline) in the pamidronate iv group. The BMD (SD) of the hip increased non significantly in the oral group with 0.008 (0.046) g/cm2 (+1.1% p = 0.358) and 0.010 g/cm2 (0.046) (+1.4% p = 0.242) in the pamidronate iv group. (table 2) There was no statistical difference between the two groups in the effect on BMD in spine (p = 0.351) or hip (p = 0.724).

**Table 2:** Change of BMD in patients treated with pamidronate iv or oral bisphosphonates

	**Oral bisphosphonates n = 31**			**Pamidronate n = 30**		
	BMD Start G/cm2 (SD)	BMD after 4.2 years treatment g/cm2 (SD)	Change of BMD % (range)	BMD Start g/cm2 (SD)	BMD after 4.3 years treatment g/cm2 (SD)	Change of BMD % (range)

Spine	0.800 (0.171)	0.856 (0.139)	8.3 (55.3)*	0.782 (0.092)	0.830 (0.112)	6.1 (33.8)*
Hip	0.714 (0.127)	0.724 (0.136)	1.1 (33.6) §	0.739 (0.126)	0.749 (0.116)	1.4 (27.8)‡

There is a significant increase in BMD of the spine in both groups (* p < 0.001) and a non significant increase in the hip in both groups. (§ p = 0.358 and ‡ p = 0.252)

## Discussion

Oral bisphosphonates have shown to effectively prevent bone loss and fractures. However, occasionally they can cause gastrointestinal complaints. Furthermore, therapy with oral bisphosphonates includes stringent requirements for fasting and posture during administration, causing inconvenience for some patients. [8] The combination of possible gastrointestinal side effects and a complex dosing regime can cause a decreased adherence, which has been recognized as a problem in the treatment of all chronic diseases.[9] Besides that, oral bisphosphonates are known for their poor bioavailability, which is reported to be as low as 0.9–1.8% for alendronate and risedronate.[10]

In case of gastrointestinal complaints, and in patients with contraindications for oral bisphosphonates as a consequence of abnormalities in the upper gastrointestinal tract, particularly the oesophagus, intravenous administration might be an attractive alternative. Furthermore, the administration is less complex for the patients and it provides certainty to the physician that patients receive their treatment. Also, bioavailability of intravenous administrated bisphosphonates is much better.

We realize that our study has limitations. We measured the effect of therapy by monitoring BMD change. Although this is a surrogate endpoint, the primary goal of treatment remains fracture reduction. However, to measure fracture reduction, large numbers of patients are required, particularly in a trial with a non inferiority design.[11] Nevertheless, there are some data that suggest that larger increases in BMD are correlated with lower fracture rates. [12-14]

Additionally, the study population may be subject to selection bias, since the patients treated with pamidronate had gastrointestinal problems. Although we can not exclude that the two groups were slightly different, there were no differences in risk factors for osteoporosis between the groups. Moreover, the pre-treatment period with oral bisphosphonates was short, thus their effect on bone mineral density during this period will be minimal.

Because of the fact that it is a retrospective study and only patients who tolerated treatment for at least two years were included, it was not possible to perform a risk benefit calculation. Nevertheless, our data clearly show that for these patients, who tolerate their treatment regimes, the response in BMD is comparable.

Recently, new data of large randomized controlled trials have become available, which showed that treatment with either zoledronate or ibandronate iv compared to placebo (three years and one year respectively) has a superior effect on BMD in postmenopausal women, and leads to fracture reduction. [15,16] However, in our study, we present data over a longer observation time (four years). Moreover, and more important, we compared the changes of BMD during an intravenous regime with that of widely prescribed oral bisphosphonates, usually regarded as first choice of treatment, instead of a comparison with placebo treatment, which was the case in the zoledronate and ibandronate studies.

In conclusion, our present study demonstrates that treatment during four years with intravenous pamidronate has a comparable effect on change in BMD as treatment with either alendronate or risedronate. We therefore suggest to consider treatment with intravenous pamidronate as an alternative treatment for patients diagnosed with osteoporosis who have gastrointestinal side effects during treatment with oral bisphosphonates or contraindications for oral bisphosphonates.

## Conclusion

The results of the present study demonstrate, that long term treatment with pamidronate iv has a comparable effect on BMD as oral bisphosphonates. In both groups the BMD of the spine increased with more than six percent, while the BMD of the hip remained stable.

## Competing interests

The authors declare that they have no competing interests.

## Authors' contributions

DE collected data, drafted the manuscript and performed statistical analysis. MV participated in the design of the study and helped to draft the manuscript. IB participated in the design of the study and helped to draft the manuscript. HD collected data. BD helped to draft the manuscript. WL conceived of the study, participated in its design and coordination and helped to draft the manuscript. All authors read and approved the final manuscript.

## Pre-publication history

The pre-publication history for this paper can be accessed here:


